# Supplementary material for: Effect of Phytochrome Deficiency on Photosynthesis, Light-Related Genes Expression and Flavonoid Accumulation in Solanum lycopersicum under Red and Blue Light
Source: Cells. 2022 Oct 31;11(21):3437. doi: 10.3390/cells11213437 (PMC9658692; doi:10.3390/cells11213437)
Supplement: Supplementary file 1 [file cells-11-03437-s001.zip › Table S1 primers.pdf]

Table S1. Primers for qRT-PCR analysis

№	Gene Bank ID	Gene description	Gene	Primer 5′-3′	
				**Forward**	**Reverse**
1	NM_001374394.1	anthocyanidin synthase	*ANS*	TCTCAATTCCCACCTCGCAC	ACTTTGCGCTCAGCAAGAAC
2	NM_001247883.2	phytoene synthase	*PSY*	CTCCATCTGGAGAACGGACG	CAACAAGCCCAAATTCCCCG
3	NM_001247104.2	chalcone synthase	*CHS*	CCGTGAACCCAGTGAATCTC	CTCACGTAGGTGTCCGTCAAT
4	NM_001247891.2	transcription factor elongated hypocotyl 5	*HY5*	AAGCTCAACCATCAGCTGGG	CCTTCACCCTTGCTTCCAGA
5	NM_001308008.1	transcription factor phytochrome interacting factor 4	*PIF4*	GAGTTGTTGTGGCGAAACGG	TAGCCACGCGACAGTTTCAT
6	NM_001247118.2	E3 ubiquitin-protein ligase	*COP1*	TGAACCTGCAGATGCACACT	AATCAACACTCCAAGCCCGT
7	XM_004249510.4	phenylalanine ammonia-lyase 1	*PAL1*	TGCAGCTCCAACTACCCTTT	ATTCGTCCTCGAAAGCTCCA
8	NM_001309257.1	chloroplastic PS II nonphotochemical quenching protein	*PSBS*	TGTTGGCCGTGTTGCTATGA	TTGCCAGGAGGGATAACAGC
9	NM_001247219.2	de-etiolated1	*DET1*	AAGCTGGCAGCACAGATGG	CTGATGCCTGCAGAAACAAGG
10	Solyc06g008030	phytochrome interacting factor 1b	*PIF1b*	TAGTATGGCAAAATGGTGGAG	CGGCGTCACAACTCGGTG
11	Solyc01g102300	phytochrome interacting factor 3	*PIF3*	AAGGCTTCCCAATAATGC	CCATCAGACCAAACTTCCC
12	Solyc06g069600	phytochrome interacting factor 7b	*PIF7*	GTACTCCACCACAACCTATT	TTGACATTTTCTTGATTGCCC
13	Q2MIC0	photosystem II D1 protein	*PSBA*	TGAAGGTTACAGATTCGGTCA	TGAATATGCAACAGCAATCCA
14	A0A0C5CUN5	photosystem II D2 protein	*PSBD*	GATATTATGGATGACTGGTTACG	CATTCTCTGGTCCCATTGC
15	P12360	chlorophyll ab binding protein 6A	*CAB6*	CTGAAGACCCGGAGGCATTT	TGCAAGGTGATCAGCAAGGT
16	Q588D6	phytochromobilin synthase	*Aurea*	ATGAAAGCTCTGGTGTTTCTTAC	CCGTCGGTCCCTTCAATACAT
17	I6QQX8	golden 2-like 1 transcription factor	*GLK1*	AGTGAAGAATCTACGCAACAAAGG	TTCTGGTGTCCAGTCCACCT
18	Solyc04g007210	B-box domain containing protein 13	*BBX13*	TGTGCATGGGAAATTGTGGG	ACCTTGTTCGTCGCTTTTCC
19	Solyc05g009310	B-box domain containing protein 15	*BBX15*	CTTCTCCAACTACTCACAATCATC	GTTCGTCACTACTTCATCGTCTCC
20	Solyc12g005750	B-box domain containing protein 16	*BBX16*	CAGAAGCCTATGTATATTGTGAAGCAG	TCATGTGAAACTCCAATCAAATACC
21	Solyc07g052620	B-box domain containing protein 17	*BBX17*	GAGCTGATTTTGCCGACGAT	CGTTCTGTAGTCCGTCGTCT
22	Solyc12g006240	B-box domain containing protein 7	*BBX7*	CGAAGGGCTGCTATATGGGA	TCAAGTCCACCGCATCCATA
23	Solyc12g089240	B-box domain containing protein 20	*BBX20*	GGATGAGGCTGCTCTTTGTG	AACCCCTCCTTTCCTGACAG
24	Solyc04g081020	B-box domain containing protein 21	*BBX21*	GCAGCTTCATGTAGTGCGAA	ACAGAACCAGAAACAGGGGA
25	Solyc06g063280	B-box domain containing protein 30	*BBX30*	GTCGACTTGTGTTTCCAGCA	TTCACCGTTAGAGTCGCTGT
26	AAL75893.1	*Solanum lycopersicum Actin* 1 Tom52	*Actin1*	TTAGCAACTGGGATGACATGGA	CCTGAATGGCAACATACATAGCA
