# Supplementary figures and images for: Effect of Phytochrome Deficiency on Photosynthesis, Light-Related Genes Expression and Flavonoid Accumulation in Solanum lycopersicum under Red and Blue Light
Source: Cells. 2022 Oct 31;11(21):3437. doi: 10.3390/cells11213437 (PMC9658692; doi:10.3390/cells11213437)

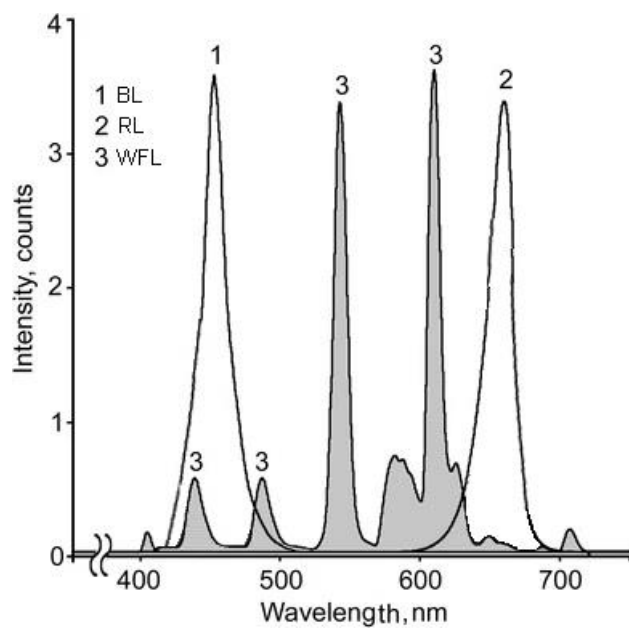

Supplementary Figure S1: Emission spectra of the light sources used in the experiments

Supplement: Supplementary file 1 [file cells-11-03437-s001.zip › Supplementary Figure S1.pdf]
